# Combined laser-based X-ray fluorescence and particle-induced X-ray emission for versatile multi-element analysis

**DOI:** 10.1038/s41598-021-86657-6

**Published:** 2021-05-11

**Authors:** Pilar Puyuelo-Valdes, Simon Vallières, Martina Salvadori, Sylvain Fourmaux, Stephane Payeur, Jean-Claude Kieffer, Fazia Hannachi, Patrizio Antici

**Affiliations:** 1 INRS-EMT, 1650 blvd. Lionel-Boulet, Varennes, QC J3X 1P7 Canada; 2 CENBG, CNRS-IN2P3, Université de Bordeaux, 33175 Gradignan Cedex, France; 3 CELIA, CNRS, CEA, Université de Bordeaux, UMR 5107, 33400 Talence, France; 4 ENEA, Via Enrico Fermi 45, Frascati, 00044 Rome, Italy

**Keywords:** Plasma-based accelerators, Ultrafast lasers

## Abstract

Particle and radiation sources are widely employed in manifold applications. In the last decades, the upcoming of versatile, energetic, high-brilliance laser-based sources, as produced by intense laser–matter interactions, has introduced utilization of these sources in diverse areas, given their potential to complement or even outperform existing techniques. In this paper, we show that the interaction of an intense laser with a solid target produces a versatile, non-destructive, fast analysis technique that allows to switch from laser-driven PIXE (Particle-Induced X-ray Emission) to laser-driven XRF (X-ray Fluorescence) within single laser shots, by simply changing the atomic number of the interaction target. The combination of both processes improves the retrieval of constituents in materials and allows for volumetric analysis up to tens of microns and on cm^2^ large areas up to a detection threshold of ppms. This opens the route for a versatile, non-destructive, and fast combined analysis technique.

## Introduction

In recent times, laser-based sources as produced by high-intensity (>10^18^ W/cm^2^) short-pulse (ps–fs) lasers in the multi-hundred TW or even PW regime, have raised interest for their manifold applications. The wide-range use of these laser-driven particle sources has triggered the construction of a series of laser facilities with dedicated laser-based beamlines (to cite a few of them: ALLS^1^ in Canada, APOLLON^2^ in France, VEGA^3^ in Spain). Among the many applications, laser-based proton beams can be utilized for producing bright ultra-short neutron sources^4^, in medicine^5^, for picosecond metrology^6^, for stressing and testing materials^7,8^, and lately also in Ion Beam Analysis (IBA)^9–12^. Within the IBA techniques, we can name the Particle-Induced X-ray Emission (PIXE), a particle-based spectroscopy technique used for retrieving the elements of a material. Other similar techniques are for instance X-ray Fluorescence (XRF) or the electron-based Energy Dispersive X-ray fluorescence (EDX).

Among all these techniques, XRF and PIXE are both well-established, non-destructive, multi-element analysis techniques, providing the most complete information about the elements of materials, in particular when coupled with backscattering techniques. They allow retrieving an exact fingerprint of a material due to the yield of characteristic X-ray emitted from the sample. Its working principle is based on the fact that when an electron is ejected from an atom's inner-shell, an electron from a higher level replaces the missing lower level electron, filling the vacancy. In XRF, the first electron is ejected by a high-energy X-ray photon while in PIXE, it is ejected by a proton or other positive ions. Both techniques are routinely used for analysis of cultural heritage^13,14^, where there is a stringent need for improved techniques^15–17^. Moreover, they are also widely used in biomedical^18,19^ or environment^20^ applications. Most studies agree that both techniques are complementary^21,22^: they have their advantages and drawbacks that depend on the sample matrix and the atomic number of the studied element^13,19,23^, and the sample size. For example, photons and charged particles have different penetration depths for objects: while XRF analytical depths are relatively large (few millimeters), PIXE analytical depths are smaller (dozens of micrometers, depending on the energy spread of the impinging particle beams), thus allowing for a layer-by-layer analysis. However, both techniques react differently regarding the background signals. In PIXE, the background intensity distribution decreases with increasing atomic number while for XRF the background noise increases with increasing atomic number^24^. Therefore, PIXE technique is better suited for relatively high atomic number elements, while XRF is better suited for elements with smaller atomic numbers. Additionally, the highest XRF fluorescence yield is obtained for photon energies located just above the absorption edge of the atom to be detected. Another difference is that for the analysis of a well-defined position in a sample, the PIXE spot-size can be easily adjusted down to microns, while this is not possible when using XRF. Unfortunately, PIXE using conventional (radio-frequency or electrostatic) accelerators requires complex and costly facilities, which leads to a lower employability. Conventional XRF technique is portable and the used X-ray energies can be tuned. However, the r^−2^ dependence of the intensity makes it very difficult to design an apparatus that allows a high lateral resolution^22^.

A laser-based XRF and electron-induced technique based on moderate laser intensities (10^16^–10^17^ W/cm^2^) has been proposed recently to explore pigment samples^25^. The laser hits onto a solid foil (target) and its atoms are ionized due to the intense electric field of the laser. Electrons are accelerated and re-injected into the target bulk material generating X-ray radiation. In the generated target spectrum, the discrete lines corresponding to the interaction target material appear on the top of the continuous Bremsstrahlung spectrum, and depend on the target's atomic number^26^. The generated X-rays and electrons can be used to probe samples.

Additionally, laser-based proton sources, requiring lasers with an intensity I > 10^18^ W/cm^2^, have been used to investigate a laser-based PIXE diagnostic (laser-PIXE), both experimentally^9,10^ and theoretically^11^. The laser-acceleration was produced using the most routinely available acceleration mechanism that tends to provide more reliability and stability for the accelerated ions, the so-called Target Normal Sheath Acceleration (TNSA)^27^. It occurs when a high-intensity short-pulse (duration < 1 ps) laser hits a target, typically a solid target in the micrometric thickness range. In this ultra-intense-laser-matter interaction, energetic electrons (“hot electrons”) are pushed from the front target surface inside the target by the laser's ponderomotive force. While some electrons manage to escape at the rear target surface due to their high kinetic energy, some remain retained at the back-target surface by the positively charged target bulk. As a result, a strong (TV/m) electric field is set up at the rear target surface interface. This electric field accelerates ions at the back surface of the initially unperturbed micrometric foil. The ions are coming from hydrocarbon impurities and contaminants located on the surface. The ions stem out of the target almost normally, with a conical divergence of about 20° half-angle and a Maxwellian energy spectrum. In addition to ions, the ultra-intense laser–matter interaction produces photons and accelerates electrons. The X-ray line emissions are almost isotropic, although in our experiment only X-rays in the direction of the proton beam are of interest.

In this paper, we show that an ultra-intense laser–matter interaction produces a versatile, non-destructive, fast analysis technique that allows, within a single sub-ns shot, to switch from laser-driven PIXE to laser-driven XRF, or to apply both techniques simultaneously. By simply changing the atomic number (Z) of the laser interaction target, one can toggle between these techniques from shot to shot, in the same installation, within seconds or less (the delay depends on the time to move from one target to the other, currently the community is targeting repetition rates > 1 Hz^28^). This versatility allows performing firstly a volumetric analysis (using the X-rays or a large energy spread of the protons) and then a layer-by-laser analysis (using narrow band proton energies). Postprocessing analysis tools allow retrieving the exact weight composition based on each single diagnostic contribution. Versatility in the analysis techniques is important in fields where enhanced diagnostic techniques are needed (e.g. in the cultural heritage^29–32^). In the following, we will name this technique Laser-based X-ray and Particle-Induced Fluorescence (laser-XPIF). The term *laser* will be omitted hereinafter to simplify the reading.

## Experimental setup

**Figure 1:**
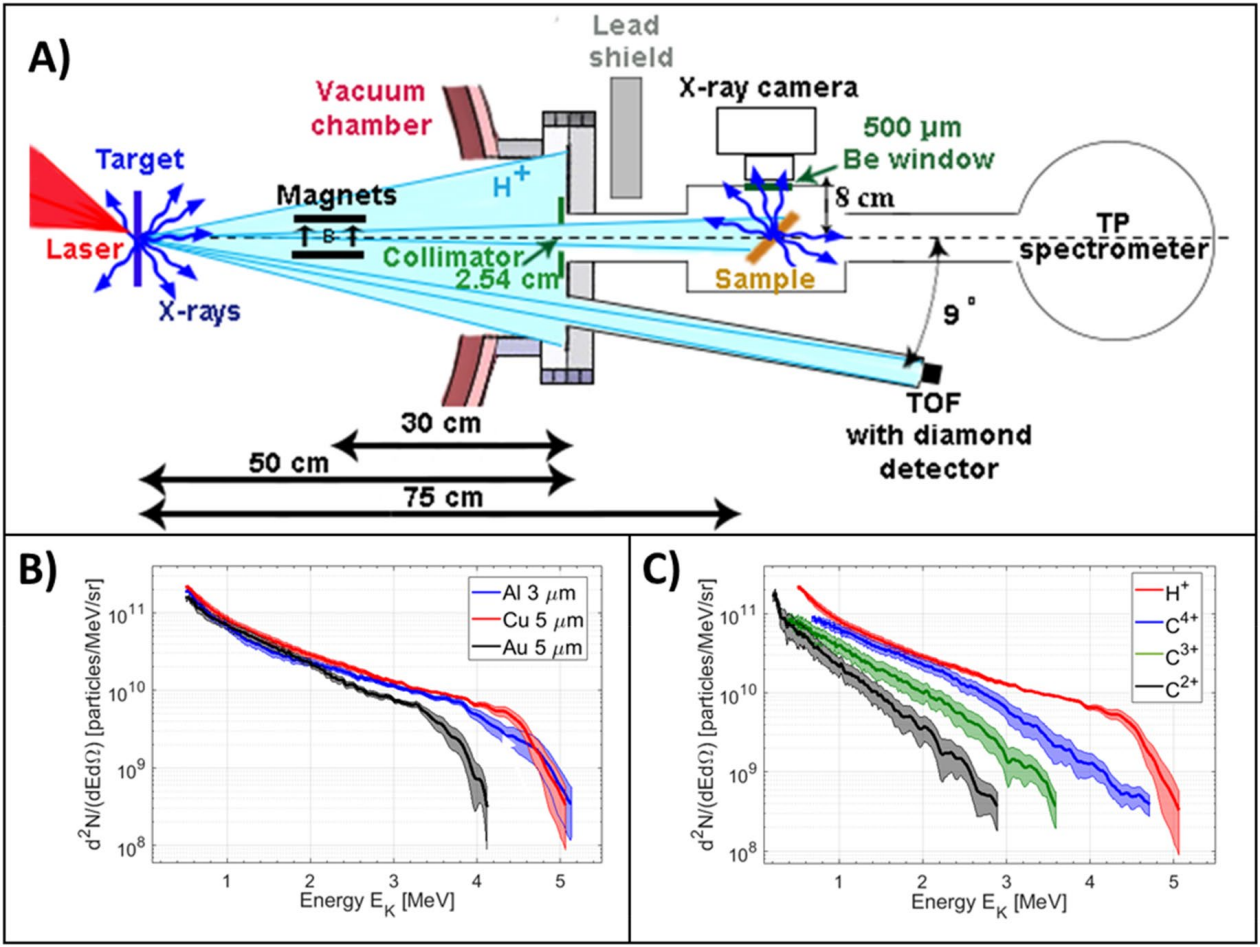
Experimental setup and ion spectra. (**A**) Experimental setup. The interaction of the 100 TW laser with the solid target (left blue) accelerates several types of ions species and generates X-rays. The ions and X-rays propagate under vacuum to the sample (orange right) to be probed, and the ion detectors. The X-rays generated by the sample are analyzed by the X-ray camera. (**B**) Proton energy spectra as accelerated by an Al 3 µm (blue), Cu 5 µm (red) and Au 5 µm (black) thickness targets, measured by the Thomson Parabola (TP) spectrometer equipped with a MicroChannel Plate (MCP) located at 0°. (**C**) Particle spectra as accelerated by a Cu 5 µm thickness target for different laser-accelerated ion species (H^+^ in red, C^4+^ in blue, C^3+^ in green, and C^2+^ in black as obtained by the TP spectrometer. Each spectrum is averaged over 10 shots and the uncertainties are calculated using the standard error of the mean.

The experiment was performed on the ALLS 100 TW laser facility located in Varennes, Canada. The Ti:sapphire laser operates at a central wavelength *λ*_0_ = 800 nm and delivers pulses with an energy of 2 J after compression in a pulse duration of 20 fs at Full-Width-Half-Maximum (FWHM). The 100 mm (at e^−2^) laser beam was focused down by an f/3 off-axis parabola to a 5 μm diameter spot (FWHM), producing an on-target intensity of about 1.3 × 10^20^ W/cm^2^ under high vacuum conditions (< 10^−6^ mbar). Using *p*-polarized laser pulses incident at an angle of 20° with respect to target normal, the laser interaction was made at best focus with three different atomic number targets, namely foils of 3 µm aluminum (Z = 13), 5 µm copper (Z = 29) and 5 µm gold (Z = 79) (purity 99.9%, purchased from *Goodfellow*). Figure 1A shows the experimental setup. The material sample to be analyzed using laser-based sources was positioned on-axis within a small auxiliary aluminum chamber connected to the main experimental chamber at 75 cm from the laser-matter interaction point. The samples were treated without any physical contact to avoid undesired signal in the X-ray spectra. The sample was oriented at 45° with reference to the proton cone-beam symmetry axis (0° axis) such as to maximize detection efficiency. In order to deflect the electrons generated during the laser-matter interaction, two magnets producing 0.1 T magnetic field at mid-distance were placed within the 0° axis at a distance of 20 cm. The presence of these magnets did not alter the proton energy distribution at 0°. In this setup geometry, the diameter of the proton beam was of 3.8 cm at the center of the auxiliary chamber, where the samples were placed. A collimator of diameter 2.54 cm was placed at a distance of 50 cm from the interaction target at 0° to avoid any interaction between the laser-based sources and the KF40 tube that connects the main chamber with the auxiliary chamber. This interaction could produce an undesired XPIF signal within our detector.

For measuring the X-ray production, a PI-LCX:1300 X-ray camera cooled with liquid nitrogen (1300 × 1340 pixels of 20 µm) was placed at a distance of 8 cm from the sample and at 90° with respect to the 0° proton axis. The quantum efficiency of the detector extended above 20 keV, allowing us to retrieve X-ray photon spectra by single-photon counting within a range from about 2.2 to 30 keV. The energy resolution of the camera can be calculated by using the Fano-limited resolution formula^33^ and yields to about 0.2 keV for 8 keV. We tested the camera by measuring X-rays of elements such as Ca (K_α_ = 3.69 keV and K_β_ = 4.01 keV), up to Ag (K_α_ = 22.16 keV and *K*_*β*_ = 24.94 keV).

The X-ray camera was placed outside the main chamber, shielded with lead bricks and far from the laser-interaction point to minimize the effect of strong Electro-Magnetic Pulses (EMP) produced during the laser-matter interaction^34^. A 250 µm thickness Be window of diameter 5.08 cm, which allows the transmission of 90% of X-rays with 8 keV energy, was used to keep the camera in vacuum, protect it from visible light and reduce the background signal. An identical window was used to keep the vacuum in the auxiliary chamber.

Different ion diagnostics were used: a Thomson Parabola (TP) spectrometer, located at 0° with respect to the ion axis, equipped with a MicroChannel Plate (MCP), as well as a Time-of-Flight (TOF) delay line equipped with a diamond detector positioned at 9°^35^. This setup was allowing the sample to be inserted (or not) inside the auxiliary chamber before every shot using gate-valve isolations along with an independent pumping system. This allowed to use either the TP or the XPIF setup on the 0° axis within a few minutes of pumping time.

Typical averaged ion spectra with their uncertainties, as obtained with the employed targets and measured using the 0° TP spectrometer are displayed in Fig. 1B exhibiting maximum proton energy of about 5.0 ± 0.5 MeV for the Cu and Al interaction target, 4.0 ± 0.5 MeV for the Au interaction target, and a mean integrated proton yield of about 2.0 × 10^11^ protons/sr with a statistical (shot-to-shot) fluctuation of 15% in the central section of the spectrum around 3 MeV, as measured over 10 shots in an identical configuration.

We employed simultaneously the TOF and TP when measuring the proton spectra. This configuration allowed to cross-calibrate the two diagnostic systems^36^ and to relate the proton spectra measured at 9° by the TOF line with the one measured at 0° by the TP spectrometer. With this configuration, we could measure indirectly the main on-axis characteristics of the proton beam impinging on the target, shot-by-shot and in real-time, even if the sample was blocking the TP spectrometer.

Concerning the other main ion species (C^4+^, C^3+^ and C^2+^) simultaneously accelerated by TNSA mechanism (see Fig. 1C), we find an integrated particle number of 8.0 × 10^10^ particles/sr for C^4+^, 6.4 × 10^10^ particles/sr for C^3+^ and 3.9 × 10^10^ particles/sr for C^2+^, all of them with a statistical fluctuation of 55%. To estimate the contribution of these heavy ions compared to protons in the PIXE process, we use the Monte Carlo simulation code called Geant4^37^, a reference toolkit for the simulation of the passage of particles through matter. The results show that the heavy-ion contribution is negligible as the particle-induced X-ray emission signal is more than eight times smaller than the proton-induced one (details can be found in the “Supplementary Materials”).

As mentioned before, X-rays are also generated from the laser-matter interaction, and each laser-irradiated target emits its own characteristic atomic spectrum^38^ besides the Bremsstrahlung background. Whenever the impinging X-ray energy is higher than the sample element-binding energy, B_K_^39^, XRF can be produced in the sample. The versatility of the XPIF technique is based on this criterion: when we consider only characteristic line emission, given the detection range of ~ 2 to 20 keV and the use of Al (Z = 13), Cu (Z = 29) and Au (Z = 79) interaction targets, we can produce XPIF signal with or without XRF contribution. In order to obtain a pure XRF contribution, it would be sufficient to simply place strong enough magnets in between the laser-interaction target and the studied sample to deviate the laser-accelerated protons from their trajectory. For low Z targets, such as Al, the X-ray lines (K_α_ = 1.49 keV and K_β_ = 1.56 keV) are not producing any XRF detectable by our diagnostic since the element with the lowest K_α_ energy observable by the imaging system is Ca and has a binding energy of B_K_ = 4.04 keV (Ca B_K_ > Al K_α_ & K_β_). Bremsstrahlung can be neglected due to its Z^2^ dependency. No XRF contribution is expected. On the other hand, for higher Z targets such as Cu, the Cu X-rays (K_α_ = 8.05 keV and K_β_ = 8.90 keV) and Bremsstrahlung can produce inner-shell vacancies in elements up to Ni (Z = 28), which has a binding energy of B_K_ = 8.33 keV. In the case of Ni, the XRF can be only induced by the Cu K_α_ or the Bremsstrahlung, both energies are above the Ni B_K_. The Cu K_α_ energy is not high enough to generate XRF with Ni elements. In the case of Au, XRF produced by L_α_ (9.71 keV), L_β_ (11.44 keV) and Bremsstrahlung is expected to contribute to the process. The higher L_α_ and L_β_ energies are able to generate XRF in heavier elements than the Cu K_α_ and K_β_.

In the experiment, to estimate the amount of atomic X-rays that induce XRF in the samples for the Cu laser-matter interaction target, we proceeded as follows:

1. The X-ray spectrum was measured by temporarily orienting the X-ray camera towards the laser-matter interaction point, for technical constraints at an angle of 6˚ with respect to the target-normal axis.
2. Rayleigh scattering of Cu K_α_ and K_β_ on pure samples (e.g. Mo, Zn and Ti) was studied using the Geant4 simulations (see details in “Materials and methods”). In the global photon calculation, the relative contributions between the subshell yield probabilities (i.e. between the K_α_ and the K_β_) were taken into account. We made the assumption that this relative contribution did not change in the plasma state generated during the laser-target interaction and used the tabulated values^40^. Geant4 simulation results were scaled to the measured number of photons in order to compare the simulation and experimental results.

**Figure 2:**
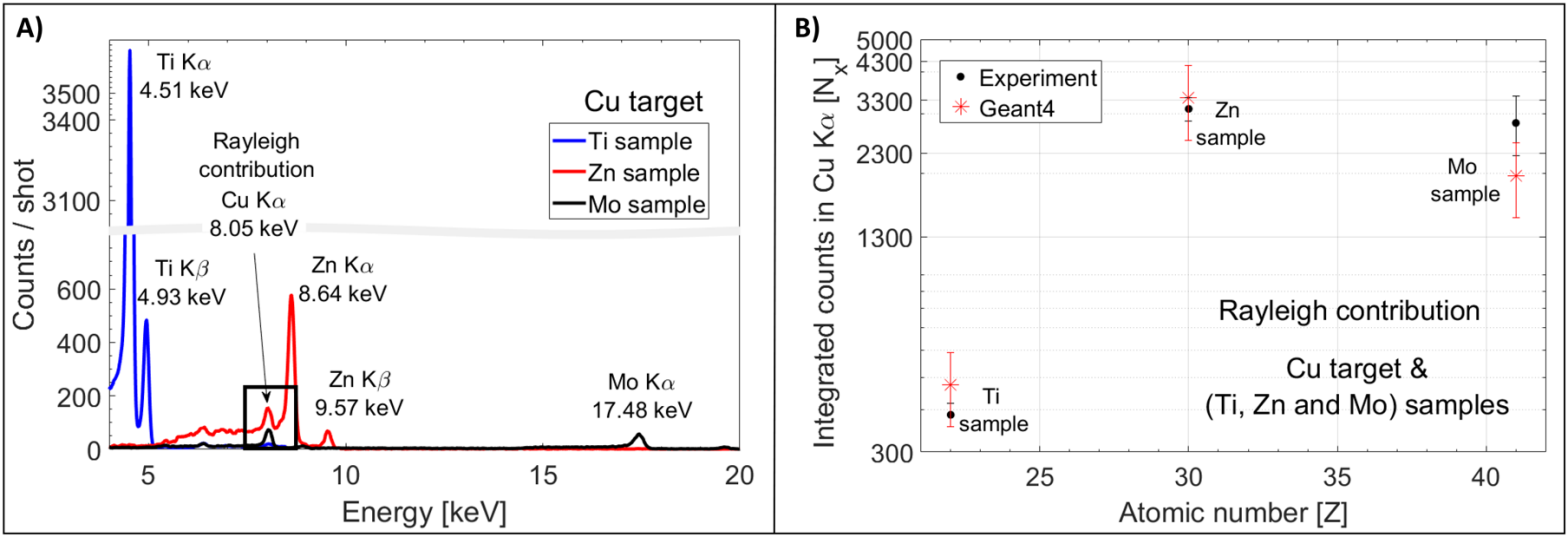
X-ray spectra and Rayleigh contribution. (**A**). X-ray spectra as obtained by the interaction of laser-based sources produced by a Cu target and a Ti, Zn and Mo sample. The Rayleigh contribution from the Cu X-rays is visible around 8 keV (see black box). (**B**) Integrated number of counts in the Cu K_α_ Rayleigh peak in Geant4 simulations when 8.05 keV photons were sent in the sample, scaled to 4.3 × 10^10^ ± 1.1 × 10^10^ incident photons/sr.

Figure 2A shows the X-ray sample spectra obtained in one single shot for Ti (Z = 22), Zn (Z = 30) and Mo (Z = 42) when irradiated by the laser-based sources produced by a Cu interaction target (details will be discussed later), while Fig. 2B shows the corresponding integrated measured number of counts in the Cu K_α_ Rayleigh peak obtained with the three material samples (black dots). The simulation results (in red asterisks) match for 4.3 ± 1.1 × 10^10^ photons/sr, which is in reasonable agreement with the measured X-ray spectrum. This allows verifying the X-ray contribution produced during the interaction.

## Results

**Figure 3:**
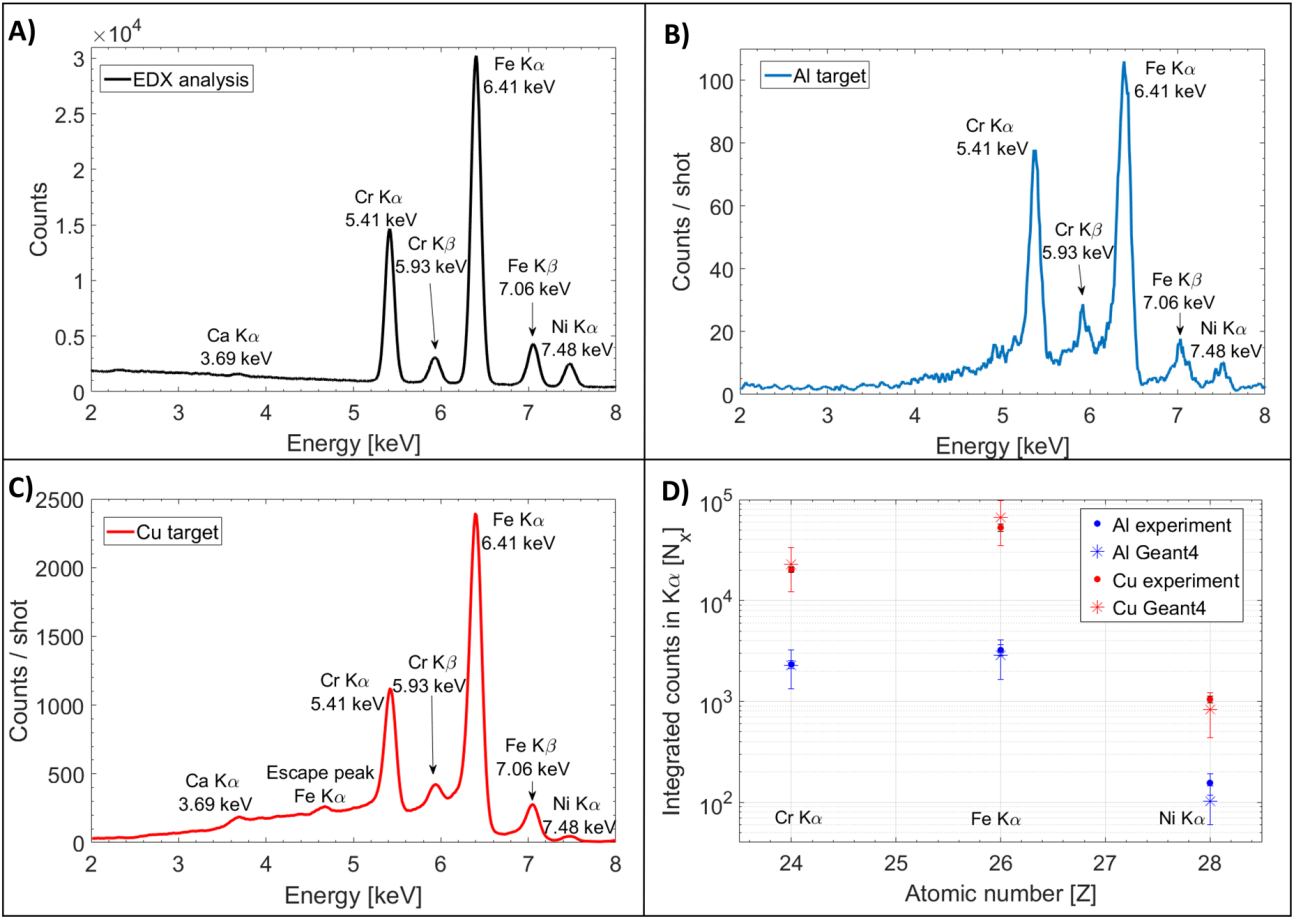
Stainless steel sample analysis. (**A**) EDX spectrum; (**B**, **C**) X-ray spectra obtained by a single shot irradiation, using the laser-based sources produced with a low Z (Al, blue) and higher Z (Cu, red) target respectively. (**D**) Measured integrated number of counts in the respective Fe, Ni and Zn K_α_ peaks (presented in dots) obtained from the spectra depicted in (**B**, **C**). Geant4 simulation results are presented with asterisks.

To study the XPIF technique and the contributions of either only protons or X-rays and protons, we irradiated a stainless steel sample (purchased from *McMaster-Carr*) and changed the laser-interaction target from Al to Cu (from low to higher atomic number). The sample size was 6 × 5 cm^2^ and it had a thickness of 1.54 mm. It had been previously analyzed using Energy Dispersive X-ray (EDX) spectroscopy, in conjunction with Scanning Electron Microscopy (SEM) (LYRA3 TESCAM). The analysis revealed the following constituents: 18.22 ± 2.87% Cr, 64.72 ± 2.92% Fe, 8.37 ± 3.11% Ni, 0.12 ± 3.84% Ca (see Fig. 3A).

Figure 3B shows the X-ray spectra obtained when irradiating in a single shot the same stainless steel sample using the source produced by an Al interaction target. This spectrum is depicting merely PIXE since line emission X-rays produced by the Al interaction target are not producing any detectable XRF. In addition, the contribution of the Bremsstrahlung produced by electrons in the Al target is negligible. One can observe the same peaks related to the elements observed by EDX, with the exception of the Ca signal that is not detected in our experiment when we were using an Al target as proton source. With an improvement of the proton spectra (an increase of the proton number and energy), we will be able to enhance the emitted X-ray yield.

By simply changing the interaction target with a higher Z target (a Cu target) there is an increase on the spectral intensity by almost 20 times (see Fig. 3C). This allows revealing the Ca element, previously not detectable. We can also observe an escape peak in Si-based detector from the Fe K_α_ at 4.66 keV. This is caused by the emission of the silicon K_α_ X-rays from the detector near-surface regions. The escape peak energy appears 1.7 keV below the primary peak and is particularly visible when the primary peak is intense. The appearance of the escape peaks can be solved by increasing the distance from the sample to the camera or placing a suitable absorber in between them to lower the X-ray flux. Since the protons spectra produced by an Al and Cu interaction-target are almost identical (see Fig. 1B), one can conclude that the increase of the photon yield is solely due to the XRF contribution.

Geant4 simulations were performed in order to confirm the relative XRF and PIXE contributions using for the material sample the same composition as obtained by EDX. The simulation results were scaled using the measured proton spectra and the number of primary atomic X-rays generated in the laser-matter interaction: in the case of Al, only protons were considered and in the case of Cu, protons and X-rays. Figure 3D compares the integrated measured counts in the K_α_ peak of the three major elements present in the sample (Cr, Fe and Ni) when the laser-interaction target is Al (blue dots) and Cu (red dots) to the corresponding Geant4 simulation results (asterisks).

One can note a good agreement between the experimental and numerical results, confirming that, depending on the type of laser-interaction target, the contribution of XRF changes. The uncertainties in the measured number of counts are mainly due to the undefined boundaries of the peaks within the spectra. The error bars in the Geant4 values, presented in asterisks, are incorporating the total uncertainty in the proton number, including the statistical fluctuation (see Fig. 1B), the absolute calibration uncertainty and the uncertainty related to the kinetic energy indetermination for the TP, all summed in quadrature.

The XPIF technique was studied using a Cu laser-interaction target for probing different pure metallic samples, including the pure (99.99%) elements Ti, Fe, Ni, Cu, Zn, Nb, and Mo (all materials purchased from *Goodfellow*). The proton and X-ray beams covered the entire surfaces of the samples. Using a Cu interaction target, when probing elements with Z < 28 the detected signal is mostly due to XRF, while for heavier elements PIXE is dominant. For all samples, we observed in one single laser shot sufficient X-ray emission to clearly allow for a fingerprint of the sample’s constitutive elements. Figure 2A shows the spectra of pure Ti, Zn, and Mo when the laser-interaction target was Cu.

The detected signal is lower for Mo than for Ti sample mainly for two reasons: firstly, the non-uniform efficiency of the camera for different X-ray photon energies, and secondly, the difference in the PIXE and XRF cross-sections. The same reasoning can be applied for the Ti and Zn signal. In Fig. 2A, the Cu Rayleigh contribution is undoubtedly observed (see the Cu K_α_ peak highlighted in the black box), which helps to estimate the number of incident photons, as mentioned above. In our setup and with our sample sizes, a single-shot irradiation provided an unambiguous readout spectrum. Several acquisitions of the same sample would have the benefit of decreasing the fluctuations in the photon counting statistics, especially if the sample had a small volume or if the irradiated surface was composed of more materials.

As next step, we tested the minimal sample size that our setup was able to detect in a single shot. We irradiated different Ti pure samples with 38 µm thickness and variable surface area sizes from 150 down to 9 mm^2^. The choice of Ti was based on the fact that the imaging detection efficiency is optimal for the energy range of Ti characteristic X-ray emission. We found a linear dependence between the integrated number of counts in the Ti K_α_ peak and the sample area, counts ranging from about 5500 ± 2400 counts/shot for surfaces of 150 ± 8 mm^2^ down to about 95 ± 50 counts/shot for surfaces of 9 ± 3 mm^2^. The limit is based on the typical peak detection criteria implemented in conventional PIXE^24^. The minimum detected quantity depends on the ratio of the area of the counts of the characteristic peak (C_P_) to the background or noise (C_B_) beneath the peak. The Minimum Detectable Limit (MDL) is usually defined as:

$$MDL = 3\surd C_{B} \left( {within \, 6\sigma \, of \, the \, peak \, distribution} \right).$$

It should be noted that the X-ray signal depends on the elements’ individual interaction cross-sections and on the amount of noise generated in the interaction that could reduce the Signal-to-Noise Ratio (SNR). Moreover, it is necessary to take precautions concerning the Rayleigh scattering and the XPIF background. The XPIF background is very often composed by undesired X-ray signal that lies within the sensitive energy detection range of the X-ray camera. For example, the iron contained in stainless steel from the chamber windows could produce parasite signal at 6.41 keV (corresponding to its K_α_), especially when low amounts of counts are coming from a sample.

**Figure 4:**
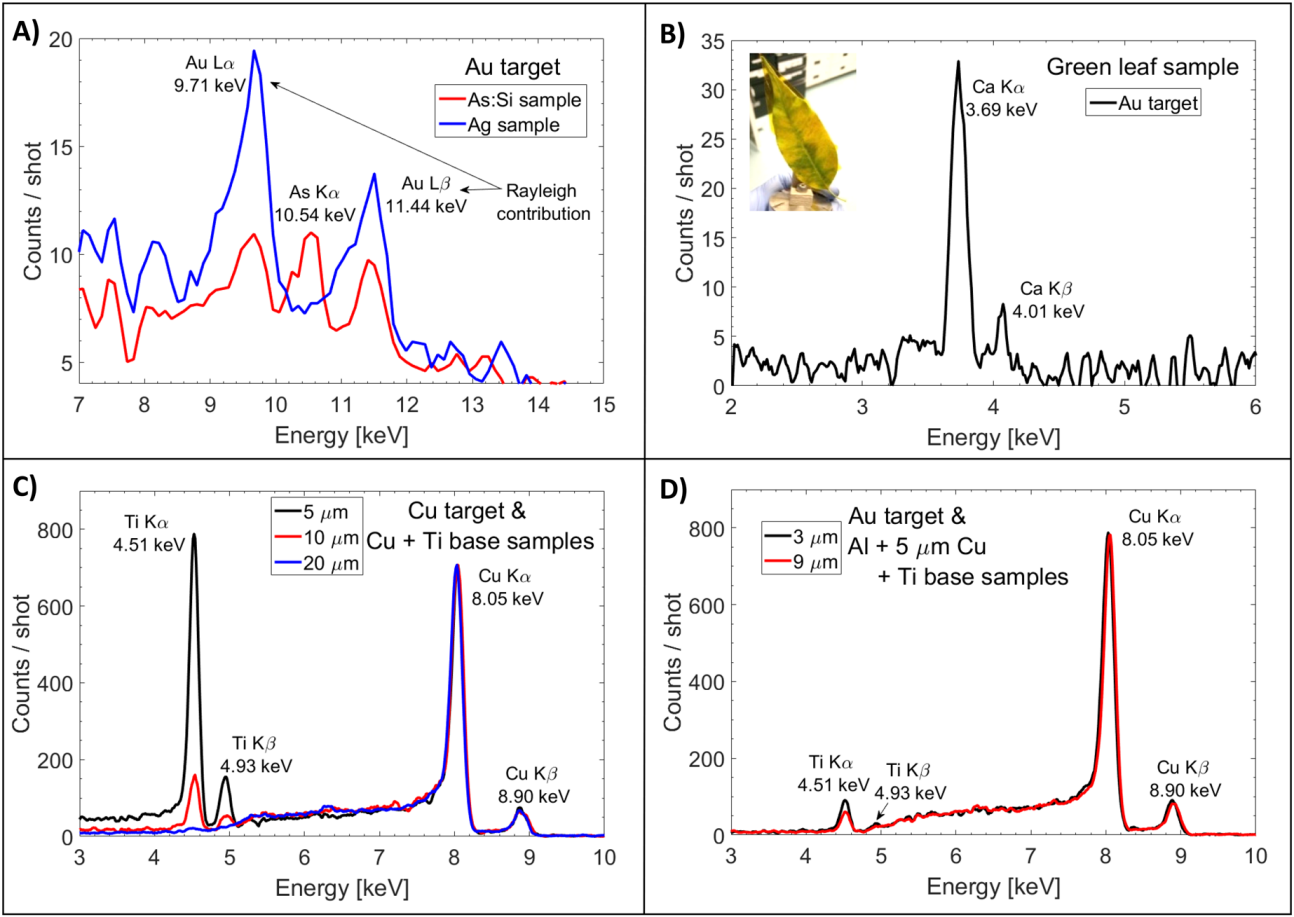
X-ray spectra obtained when irradiating different samples using the laser-based sources produced with an Au or Cu laser-interaction target. (**A**) Arsenic doped Si wafer sample (red) compared with an Ag sample (Au interaction target). (**B**) green leaf sample (Au interaction target). (**C**) 5, 10, and 20 µm Cu layer on a Ti substrate (Cu interaction target). (**D**) 3 and 9 µm Al layer lying on a 5 µm Cu layer on a Ti substrate (Au interaction target).

In order to test the minimum detectable percent composition of a sample, we irradiated an Arsenic-doped silicon wafer (As:Si) of 0.5 mm thickness and 5 cm diameter with a doping level of 20 ppm, i.e. 0.002% (supplier *WaferPro*). To be able to optimize the analysis of elements with a Z > 28, we replaced the Cu interaction target with an Au target. We observed that the resulting XPIF signal is similar (same yield and ratio between the different peak intensities) to the one obtained with Cu target for elements with Z < 28. As shown in Fig. 4A (red line), it is possible to distinguish the Arsenic K_α_ peak (10.54 keV), located in between the Rayleigh signal produced by the Au L_α_ and L_β_. To ensure that the two peaks nearby the peak located at 10.54 keV are due to Rayleigh signal, we compared the As:Si wafer spectrum with an Ag sample spectrum (blue line). We see that the Rayleigh scattering peaks due to the Au lines are still present. For the Ag sample, the peaks are higher than for the case of the As:Si wafer since the Rayleigh scattering cross-section is larger^41^. We are able to detect elements (in this case Arsenic) at least down to a level of 20 ppm.

We tested the efficiency of the XPIF technique also on non-metallic samples. Figure 4B shows the spectrum obtained by a single irradiation of a watered green leaf with a surface of about 13 cm^2^ (thickness 0.7 mm) coming from a ficus tree. In the spectrum, we can clearly see a fingerprint of Ca inside the sample, which is typical for green plants^42^.

One of the advantages of the XPIF technique is the volumetric probing: it can analyze a depth up to few millimeters if using the XRF contribution and down to several micrometers using PIXE. Figure 4C shows the X-ray spectra as obtained when irradiating three different stacks using the laser-based sources produced by a Cu target. We used two-material stacks consisting respectively of a 5, 10, and 20 µm thickness pure Cu foil placed in front of a Ti substrate (thickness 0.5 mm). The surface of all stacks was 2 × 2 cm^2^. One can identify a clear fingerprint of titanium's K_α_ and K_β_ lines up to a Cu foil thickness of 10 µm, confirming the volumetric analysis of the sample. The Ti X-rays are attenuated by the Cu sample depending on its thickness and are almost fully attenuated for a thickness of 20 µm.

We validated the volumetric XPIF also using stacks of three elements (Al, Cu and Ti). Figure 4D shows the X-ray spectra using Au laser-interaction target. This time, the stacks were formed of a 3 or 9 µm Al thickness sample, on top of a 5 µm Cu sample followed by a Ti substrate (thickness 0.5 mm). The fingerprint of Al could not be detected by the camera since its K_α_ (1.49 keV) is not in the detection range (minimum threshold value of 2.2 keV). However, one can clearly observe the elements Ti and Cu for both cases, i.e. when covered by a 3 and 9 µm thickness Al foil.

Finally, as real-setting application of volumetric XPIF on compound samples, we analyzed different metallic coins. The first coin was a Canadian quarter (25 cent, mint 2009, Nickel-plated steel; 94% steel, 3.8% Cu, 2.2% Ni plating, diameter: 23.88 mm; thickness: 1.58 mm). The coin is made of several material layers, the external layer is 5 µm Ni, which follows a 5 µm Cu layer, on top of a 5 µm Ni layer, before reaching the steel bulk. The second coin was an American penny (1 cent, mint 2000, diameter: 19.05 mm, thickness: 1.52 mm, copper-plated zinc 97.5% Zn, 2.5% Cu). The American penny is made of a 20 µm copper plating over a zinc core. As last coin, we irradiated an ancient Roman coin (Licinius I, Nicomedia mint 311-317 AD, bronze follis, 21.5 mm diameter, 3.41 g).

**Figure 5:**
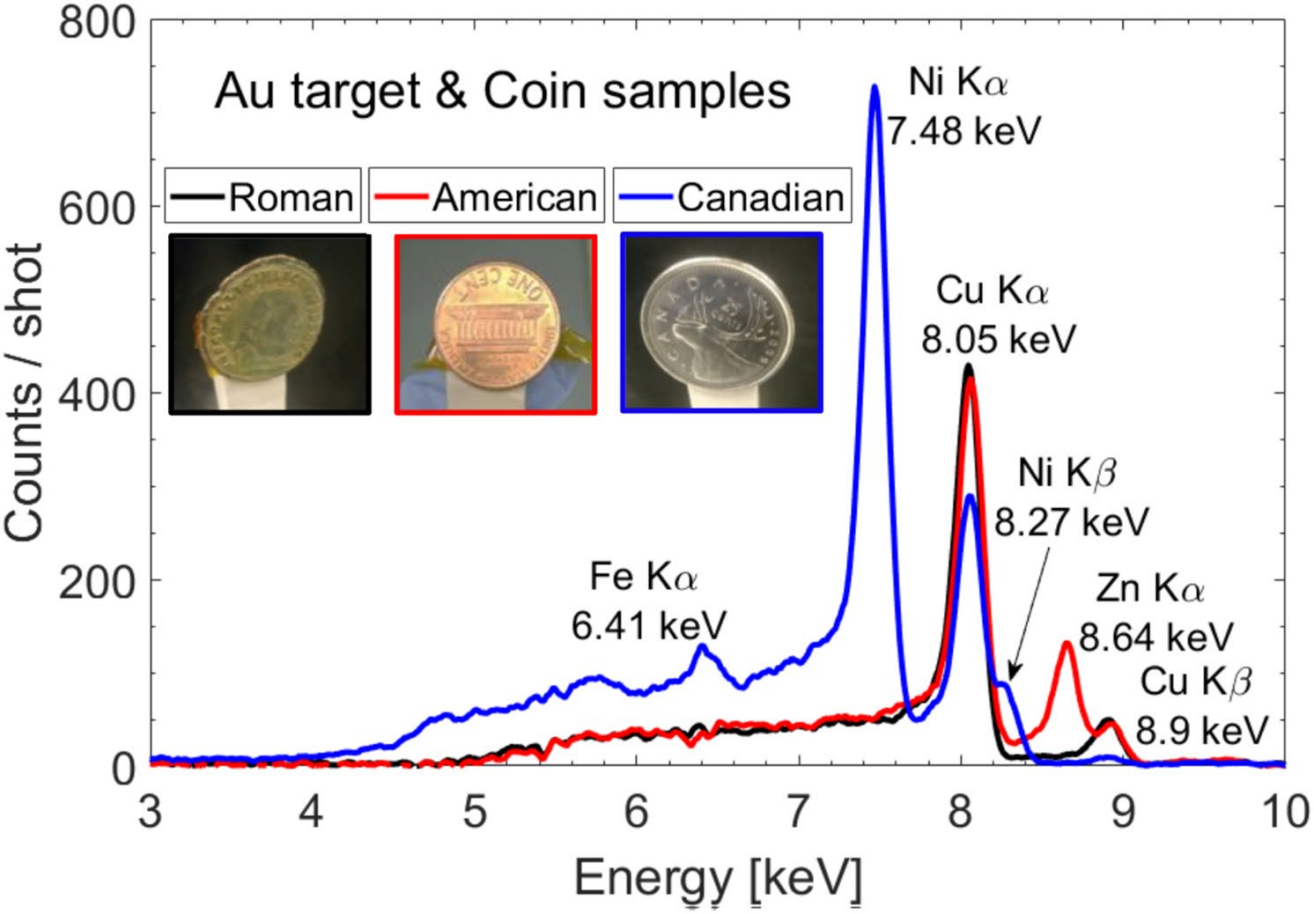
Volumetric XPIF on metallic coins. X-ray spectra obtained when irradiating a Roman (black), American (red) and Canadian (blue) coin sample using the laser-based sources produced with Au target.

The results are shown in Fig. 5. Concerning the Canadian quarter (blue line), one can clearly identify the peaks related to the constituting elements of the coin, including the main element of steel, iron. The second element contained in the alloy steel, i.e. carbon, is unfortunately not detectable by our diagnostic. Similarly, the second spectrum related to the American penny (red line) unambiguously reveals peaks related to the elements Cu and Zn, as expected. Finally, the spectrum related to the ancient Roman coin (black line) reveals the element Cu, bronze being an alloy consisting primarily of copper (~ 90%) and tin (Sn) (10%). Unfortunately, the element Sn is not detectable by our diagnostic, its K_α_ = 25.27 keV and our upper limit is 25 keV, and the L-lines are too attenuated by our imaging system.

One can notice that, in the case of the Canadian coin, the Ni K_α_ peak is higher than the other peaks, even if there is only 2.2% Ni contribution in the coin. This is because the X-rays from Ni are not attenuated by any surface layer. One can clearly assess that the XPIF is able to probe low Z elements within tens of micrometer thickness and this within a single laser shot.

As demonstrated above, laser-matter interaction allows producing either PIXE or XRF or even both, depending on the need. The combination of both enhances the detection of elements. Cumulating over several shots, or moving the sample closer to the source, allows improving the signal-to-noise ratio. Rotating or raster solid targets allow for a fast target change within seconds or faster^43^. High-repetition-rate gas-jet targets^44^, cryogenic targets^45^ are suitable for improving the PIXE signal over repetitive shots or higher particle yield. A further extension will be to study the use the technique *in-air* (air-XPIF) on more delicate samples.

Up to currently, our technique allows for a qualitative study of the constituents. Quantitative analysis requires the exact knowledge of the material’s response to the different X-rays and impinging particles. This post-processing is already performed in diagnostics based on conventional sources. The complicating factor with our technique is that our sources have a broad energy spectrum, which requires a discretization with a large number of degrees of freedom in order to be handled. This is currently under investigation. Moreover, the reliability of our XPIF depends strongly on the repeatability of the laser-based source. A precise knowledge of these sources is the key to the success of this technique. This can be obtained by measuring either real-time the laser-based sources or by lowering the fluctuations in their error bars and cumulating several shots to increase the statistics. Strong effort is performed by the community and industry to stabilize these sources.

## Conclusion

We have shown that the interaction of a high-intensity laser with a solid target generates sources of X-rays and protons that can be used for material analysis. It produces laser-based X-ray and Particle-Induced Fluorescence (laser-XPIF). By simply varying the atomic number of the laser interaction target, one can produce laser-driven PIXE, laser-driven XRF or the combination of both. Both techniques can be performed in the same installation within seconds or lower (depending on the target replacement system). The use of both techniques at the same time improves the element detection of materials. Moreover, the cross-comparison of both diagnostics in the same experimental environment enhances the reliability of the results.The quantitative analysis can be performed using post-processing tools such as implemented on conventional XRF and PIXE diagnostics. In the case of PIXE, one has to take into consideration the contribution produced by a larger ion (proton) energy spectrum, since laser-driven protons exhibit a broadband spectrum. When using both sources simultaneously, it is important to consider the contribution of both sources, which adds some complexity to the post-processing.

## Materials and methods

### X-ray camera

To measure the X-ray energy spectrum a deep-depletion X-ray camera was used. The precise measurement of the X-ray spectrum was done by photon counting^46^ as the camera has 1.74 Mega-pixel independent silicon layer detectors. If a Single Photon Event (SPE) is detected, a number of counts, N_C_, is obtained. SPE are events in which the charge is deposited only in a single pixel, with no charge spreading over the adjacent pixels. The SPE events have a sharp energy resolution, as the reading noise comes from only one pixel. The photon energy is found knowing that I_C_ is proportional to ħω where I_C_ is the channel intensity value and ħω the photon energy.

The pixel values are read at a 100 kHz frequency in order to minimize the readout noise.

### Geant4 simulations

We used the Monte Carlo code Geant4 with the low energy emlivermore Physics list^37^ to estimate the X-ray spectra resulting from the interaction of the proton and X-ray beams with the XPIF samples. We reproduced in the simulation the exact geometry of the detection set-up, including the size of the sample, its position relative to proton/X-ray source and the CCD camera, the description of the camera components and filters. Geant4 particle tracking CUTS were set to 1 µm. The size of the beam on the target sample was defined by a collimator of 2.6 cm diameter placed 50 cm from the laser interaction target. The proton spectrum was simulated by using multiple monoenergetic proton beams (with 10^7^ protons) at energy steps of 500 keV from 1 to 5 MeV. The results of the simulation were normalized to the number of protons contained in the energy distributions measured during the experiment. The interactions of Cu K_α_ and K_β_ X-rays with the samples were simulated to estimate the number of X-ray emitted in the laser interaction.

## Supplementary Information

Supplementary Figure S1.

## Data Availability

Data are available upon request from the corresponding author.
