# Supplementary material for: Combined laser-based X-ray fluorescence and particle-induced X-ray emission for versatile multi-element analysis
Source: Sci Rep. 2021 May 11;11:9998. doi: 10.1038/s41598-021-86657-6 (PMC8113557; doi:10.1038/s41598-021-86657-6)
Supplement: Supplementary file 1 — Supplementary Figure S1. [file 41598_2021_86657_MOESM1_ESM.pdf]

Combined laser-based X-ray fluorescence and particle-induced X-ray emission for versatile multi-element analysis

Pilar Puyuelo-Valdes[1,2], Simon Vallières[1,3], Martina Salvadori[1,4], Sylvain Fourmaux[1], Stephane Payeur[1], Jean-Claude Kieffer[1], Fazia Hannachi[2] and Patrizio Antici[1,*]

[1] *INRS-EMT, 1650 blvd. Lionel-Boulet, Varennes (QC) J3X 1P7, Canada.*
[2] *CENBG, CNRS-IN2P3, Université de Bordeaux, 33175 Gradignan cedex, France.*
[3] *CELIA, CNRS, CEA, Université de Bordeaux, UMR 5107, 33400 Talence, France.*
[4] *ENEA, Via Enrico Fermi 45, Frascati, 00044 Rome, Italy.*

*antici@emt.inrs.ca

Supplementary Materials

Influence of the different laser-accelerated ion species in the PIXE process

The ion spectrum obtained during the shots on the 100 TW laser facility consists of protons and other heavier ions (C^{2+}, C^{3+} and C^{4+}) that co-propagate with the ions. Using Geant4 simulations, we estimated the contribution of these heavy ions compared to protons in the PIXE process. Fig S 1 displays the Geant4-simulated number of counts in the Cr, Fe, and Ni K_α peak when irradiating the stainless steel sample with 3 MeV protons (blue) and 3 MeV carbon ions (red). The obtained values were then scaled with the measured number of 3 MeV protons and carbons. One can observe that the contribution of the carbon is eight times smaller than the proton-induced PIXE signal. The results show that the heavy-ion contribution is negligible.

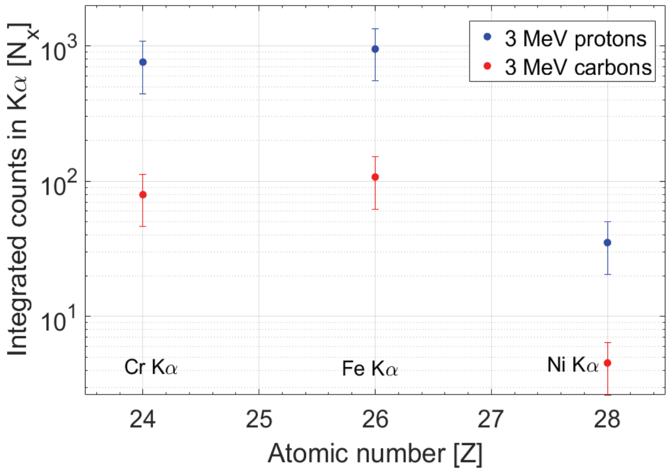

Fig S 1 Influence of the different laser-accelerated ion species in the PIXE process. Geant4 simulated number of counts in the Cr, Fe and Ni K_α peak when irradiating the stainless steel sample with 3 MeV protons (blue) and 3 MeV carbons (red) and scaled with the measured number of 3 MeV protons and carbon ions in the experiment.
